# Separating Fusion from Rivalry

**DOI:** 10.1371/journal.pone.0103037

**Published:** 2014-07-23

**Authors:** Stefan M. Kallenberger, Constanze Schmidt, Peter Dechent, Clemens Forster, Nicole von Steinbüchel, Torsten Wüstenberg, Hans Strasburger

**Affiliations:** 1 Department of Physiology and Pathophysiology, Friedrich-Alexander-Universität Erlangen-Nürnberg, Erlangen, Germany; 2 Department of Medical Psychology and Medical Sociology, Georg-August-Universität Göttingen, Göttingen, Germany; 3 Department of Cognitive Neurology, Georg-August-Universität Göttingen, Göttingen, Germany; 4 Department of Psychiatry and Psychotherapy, Charité-Universitätsmedizin Berlin, Charité Campus Mitte, Berlin, Germany; 5 Institute of Medical Psychology, Ludwig-Maximilians-Universität München, Munich, Germany; University College London, United Kingdom

## Abstract

Visual fusion is the process in which differing but compatible binocular information is transformed into a unified percept. Even though this is at the basis of binocular vision, the underlying neural processes are, as yet, poorly understood. In our study we therefore aimed to investigate neural correlates of visual fusion. To this end, we presented binocularly compatible, fusible (BF), and incompatible, rivaling (BR) stimuli, as well as an intermediate stimulus type containing both binocularly fusible and monocular, incompatible elements (BFR). Comparing BFR stimuli with BF and BR stimuli, respectively, we were able to disentangle brain responses associated with either visual fusion or rivalry. By means of functional magnetic resonance imaging, we measured brain responses to these stimulus classes in the visual cortex, and investigated them in detail at various retinal eccentricities. Compared with BF stimuli, the response to BFR stimuli was elevated in visual cortical areas V1 and V2, but not in V3 and V4 – implying that the response to monocular stimulus features decreased from V1 to V4. Compared to BR stimuli, the response to BFR stimuli decreased with increasing eccentricity, specifically within V3 and V4. Taken together, it seems that although the processing of exclusively monocular information decreases from V1 to V4, the processing of binocularly fused information increases from earlier to later visual areas. Our findings suggest the presence of an inhibitory neural mechanism which, depending on the presence of fusion, acts differently on the processing of monocular information.

## Introduction

In everyday life, most of the information from the two eyes is fused, giving rise to a unified percept. However, when incompatible stimuli are presented dichoptically, binocular rivalry occurs [1], [2]. The percept then alternates between the two images or between varying parts of one of the two [3]. Unlike fusion, binocular rivalry has been extensively studied. Binocular rivalry is considered to result from a distributed process, causing perceptual suppression or dominance of one of the two rivaling stimuli. Processes in V1 or the lateral geniculate nucleus (LGN) are most likely causing binocular rivalry [4], [5]. Besides these core regions, contributions from higher cortical areas were also shown [2], [6]–[9]. Lee and Blake [10] found that the brain response in the visual system to rivaling gratings is larger than to a condition of complete suppression, and smaller than to a condition of no suppression. Moradi and Heeger observed weaker BOLD responses to fused than to rivaling grating stimuli in areas V1–V3, and explained these by a model of inter-ocular contrast normalization [11].

Little is known about the relationship of visual fusion and binocular rivalry. They are seen as the outcome of separate processes [12] that operate simultaneously and inhibit each other [13]. Recordings of visually evoked potentials (VEP) during the presentation of fusible, rivaling, or monocular grating stimuli suggest that separate populations of neurons respond to monocular and binocular information [14] and that the switch from rivalry to fusion is not only a spatially but also a temporally distinct process [15], [16].

We aimed to disentangle the neural correlates of fusion and rivalry. To this end, we used a paradigm with a third condition (termed BFR), containing both fusible and non-fusible pattern elements. In this combined condition, one eye is presented with superimposed straight and tilted grids, while the other is presented with either straight or tilted grids only. Whereas only the two corresponding grids can be fused, all three grids are stably viewed without perceptual alternations. Perceptually, the incompatible grid appears to be situated in-plane with the fused ones. Thus, by comparing a standard condition of rivalry (BR) with the combined condition BFR, we addressed the effect of fusion. In turn, by comparing the combined condition BFR with the fusion-only condition (BF), we were able to address the effect of the additional, potentially rivaling monocular information on the fused percept.

Previous fMRI studies on fusion and rivalry did not account for visual eccentricity. However, peripheral vision seems to play an important role in these processes [17], [18]. Alternation in rivalry does not involve the visual field as a whole but happens in “zones of rivalry”. The sizes of these zones increase linearly with eccentricity according to cortical magnification [18]. Ogle and Schwartz found a comparable relationship for the Panum’s area, with a shallow but steady rate increase in diameter (0.1° diameter per 5.6° visual angle) [19], [20]. Also, in cases of impaired vision, it can be peripheral rather than foveal fusion that maintains eye alignment [21]–[23]. To address the – so far underestimated – role of the visual periphery in fusion, a second aim of our study was to analyze how the visual field outside the fovea contributes to neural processes of fusion and rivalry. In this context, we were specifically interested in the distribution of these neural processes across retinotopic maps for the central, 16°-diameter visual field.

## Materials and Methods

### Subjects and pre-examinations

Ten subjects took part in the study (3 male, 7 female), aged between 18 and 36 years (median 25.5 years). All subjects had normal vision without correction when tested with a standard eye chart and normal stereo vision (Titmus test).

### Ethics Statement

All participants gave written informed consent. The trial was conducted in accordance with the Declaration of Helsinki, after a positive vote of the ethics committee of the Medical Faculty at the University of Göttingen.

### Stimulus presentation

Stimulus presentation and timing were realized using the software *Presentation* (Version 9.00, Neurobehavioral Systems, Inc., Albany, CA, USA, http://www.neurobs.com). Into the magnet, stimuli were presented with MR-compatible LCD goggles (Resonance Technology, Northridge, CA, USA) covering a visual field of 24°×32° visual angle at a resolution of 600×800 pixels and a maximum luminance of about 70 cd/m^2^. Selective monocular stimulation was achieved by independently controlling the displays of the employed goggles using two time-synchronized computers.

#### Retinotopic mapping

Black-and-white checkerboard wedge and ring stimuli, presented at a contrast-reversal frequency of 8 Hz were used for retinotopic mapping [24]–[26]. A run started with horizontal or vertical wedges (opening angle 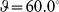; check sizes: 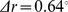, 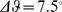; vertical wedges’ radius 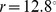 visual angle, horizontal wedges 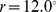). Eight functional MR volumes (2 sec) were acquired for each wedge with a total duration of 16 seconds. The wedges were followed by five annulus checkerboard stimuli of increasing radius and width, (0.8°–1.7°, 1.8°–3.1°, 3.2°–4.8°, 4.9°–6.9′°, 7.3°–9.9°, check sizes: 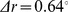, 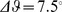), again with eight (2-sec) volumes each. A session consisted of three runs with seven stimuli, resulting in a total length of 3×7×16 = 336 seconds. Two sessions of such meridian mappings were conducted.

#### MT+ localizer

To localize area MT+, a motion mapping stimulus was designed, comprising a field of white moving random dots on a 16°×16° grey background. Random dots (n = 200) of 0.23°×0.23° size moved, in random direction, centrifugally from the central fixation cross (0.70°×0.70°) at a speed of 6.25° per second. In one run, eight static stimuli alternated with eight moving-dot stimuli of five sec duration each. Two sessions of five runs, each lasting (16+16)×5 = 160 seconds per session, were carried out.

#### Visual fusion and binocular rivalry

**Figure 1:**
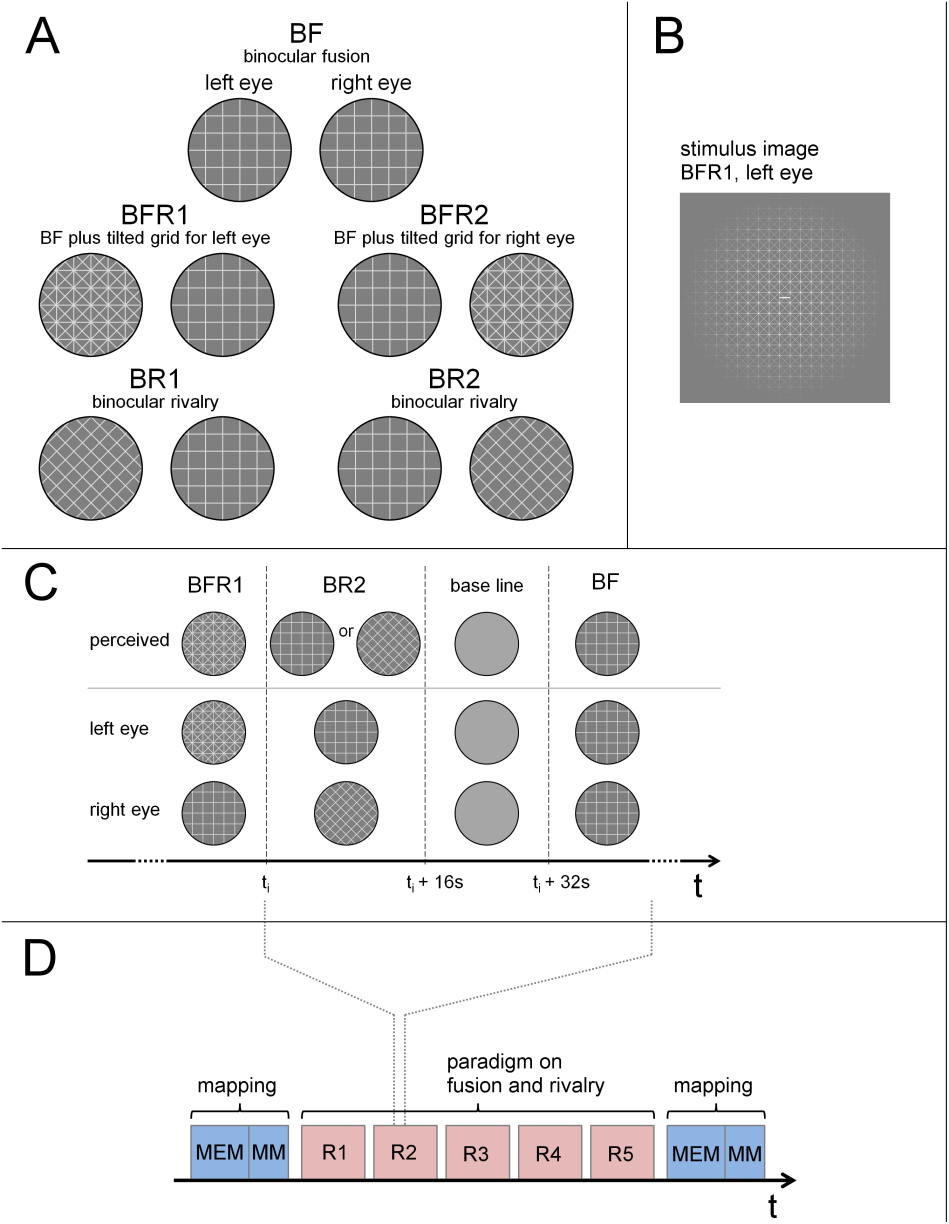
Stimulus patterns and experimental design. (A) Patterns used for the left- and right-eye stimuli, respectively, in the conditions fusion (BF), fusion with an incompatible grid present (BFR1, BFR2), and rivalry (BR1, BR2). Whereas the grid stimuli in BF can be fused, the incompatible grids in BR1 and BR2 induce binocular rivalry. In BFR1 and BFR2 (which represent a combination of BF, and either BR1 or BR2, respectively), fusion precedes over rivalry due to the additional fusible grid. (B) An actual stimulus image presented in BFR1 to the left eye as an example, in which the pattern is modulated by a Gaussian aperture. (C) Timeline of the presentation, showing an excerpt of one run. Within the run, all stimuli for the left and right eye (BF, BR1, BR2, BFR1, and BFR2) were presented in pseudo-random order. The underlying patterns of the stimulus images (i.e., without Gaussian) are shown in the two rows above the time arrow; the top row shows the resulting percept. An image with intermediate grey, representing a null stimulus, was used as baseline. We compared the summed response to BFR1 and BFR2 (termed BFR) with the sum of BR1 and BR2 (termed BR), to address the effects of inhibiting rivalry by inducing fusion, while still presenting similar stimulus elements. The comparison of BFR with BF addressed the effects of the additional incompatible monocular content during fusion. (D) After a session of meridian and eccentricity mapping (MEM) and motion mapping (MM), five runs (R1–R5) were conducted with stimuli of the paradigm on fusion and rivalry, followed by another session of meridian, eccentricity, and motion mapping.

Stimuli for fusion and rivalry were constructed by superimposing either identical or 45°-tilted dichoptic grid stimuli (Figure 1). The condition BFR was constructed by adding a monocular tilted grid to fused horizontal-vertical (upright) grids. The rationale for that derives from Blake & Boothroyd’s (1985) finding that the presence of matching features in otherwise incompatible monocular stimuli prevents the latter’s binocular suppression [27]. Thereby, the monocular tilted grid in BFR is permanently perceived, together with the fused upright grids. To balance the effects of stimulus content, identical and tilted grids were combined in various ways to allow for their changing between conditions of binocular fusion (BF), binocular rivalry (BR), and fusion with an incompatible monocular grid present (BFR). For condition BF, identical grids were shown to the two eyes. For rivalry in condition BR, a tilted grid was presented to the one eye and an upright grid to the other eye. In the combined condition BFR, a combination of an oblique and an upright grid was presented to the one eye, and an upright grid to the other.

To balance for effects that could arise from different processing of left- or right-eye information, as might be expected from eye dominance, we used two opposite spatial arrangements for BR and BFR, in which the stimulus containing the tilted grid was presented either to the left (BR1, BFR1) or to the right eye (BR2, BFR2). Therefore, BFR1 and BFR2 were equivalent to BF plus a tilted monocular grid in the left or right eye, and equivalent to BR1 and BR2 plus an additional upright grid in the eye presented with a tilted grid.

To further monitor visual suppression, a control stimulus was inserted in the stimulus center, comprising a horizontal bar for the left, and a vertical bar for the right eye. This object does not prevent rivalry in the BR condition, but can be fused to a single cross-shaped percept in the fusible conditions (BFR and BF). Subjects were instructed to fixate and to attend this control object.

Stimuli covered an area of 16°×16° visual angle and were composed of white lines of 0.04° thickness with a between-line-distance of 0.8° on mean-grey background. Sharp-edged lines were used instead of sinusoidal gratings to avoid local effects of superposition. To minimize edge effects that might interfere with fusion or rivalry, the grey value *g* of the stripes declined from 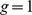 in the center at 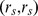 by a two-dimensional Gaussian.
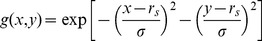
until it reached the background value of 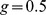 at a stimulus radius of 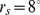 (the width given by 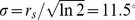). The function resulted in a (Michelson) contrast of 33% in the center, which declined to below 10% at a radius of 6.7°. These patterns can be considered as high-contrast stimuli with regard to the evoked BOLD response [11], [28] inducing a rapid switch between fusion and rivalry (cf. Text S1 and Liu et al. [29]). As measured in a pilot experiment with our stimulus set, switches from fusion to rivalry (in the transitions from BF or BFR to BR) and vice versa (in the transitions from BR to BF or to BFR) occurred in less than a second.

#### Experimental course

To verify their expected appearance and the perceptual switching, each stimulus was presented to each subject while located inside the scanner before starting the fMRI experiment. After scanning, all subjects confirmed perceptive switches via verbal report. Sessions of retinotopic mapping and the MT+ localizer were conducted before and after the fusion/rivalry paradigm. Each stimulus block in the fusion/rivalry experiment lasted 16 seconds. Blocks with BF, BR1/2 as well as BFR1/2 stimuli were presented in a randomized order, followed by a sixth block with an intermediate-grey background, serving as low-level baseline in the analysis. In one experimental session, each block occurred four times. Five such runs, each lasting 6×4×16 = 384 seconds, were conducted.

### FMRI data acquisition and stimulation setup

Data were acquired on a 3T whole-body scanner (Magnetom TIM Trio, Siemens Healthcare, Erlangen, Germany) with an 8-channel phased-array head coil. Whole-head Blood Oxygenation Level Dependent (BOLD) fMRI images were acquired using a T2*-weighted gradient-echo echo-planar imaging (GE-EPI) pulse sequence (time to repeat (TR) = 2000 ms, time to echo (TE) = 33 ms, flip angle = 70°, 27 slices, matrix size 64×64, voxel size 3×3×3 mm^3^). An fMRI session consisted of five experimental cycles: (1) a first retinotopic mapping, comprising 168 images, (2) a first MT+ localizer, comprising 80 images, (3) the fusion/rivalry paradigm, comprising 960 images, (4) a second retinotopic mapping, comprising 168 images again, and (5) a second MT+ localizer, comprising also 80 images. Thus, during the whole functional MRI experiment, we acquired a total of 1456 brain images. For spatial normalization, cortex extraction and flattening, a T1-weighted structural dataset of the whole head with an isotropic resolution of 1 mm^3^ was acquired (3D Turbo FLASH sequence, TR = 1950 ms, TE = 3.93 ms).

### Image analysis

Image processing and statistical analyses were carried out by means of Statistical Parametric Mapping software (SPM5, Welcome Department of Imaging Neurosciences, London, UK, http://www.fil.ion.ucl.ac.uk/spm/) and Caret 5.51 (Washington University School of Medicine, St. Louis MO, USA, http://brainvis.wustl.edu/wiki/index.php/Caret) [30].

*Preprocessing:* First, functional images were motion-corrected and a mean functional image was computed. Second, the individual structural image was coregistered to this mean EPI-image, segmented, and spatially normalized using a unified segmentation approach as implemented in SPM5 [31]. Third, transformation parameters as obtained in the second step were applied to the motion-corrected functional images. Fourth, the normalized functional images were spatially low-pass filtered using an isotropic Gaussian kernel of 8 mm full width at half maximum. Finally, the cortical surface was reconstructed and flattened using Caret 5.51.

*Individual voxel-wise General Linear Modeling (GLM, SPM5):* In line with the blocked experimental design, stimulus-dependent neural activity was modeled by boxcar functions and convolved with the canonical hemodynamic response function (HRF) provided by SPM. The resulting time courses were down-sampled for each sampling point to create regressors in a General Linear Model. The GLM contained separate regressors for the conditions of interest: (1) vertical and horizontal wedges and five rings with differing eccentricity for the retinotopic mapping, (2) stationary and moving dots for the motion mapping, and (3) BF, BR1, BR2, BFR1 and BFR2 for the fusion and rivalry paradigm. To capture residual fluctuations in the MR signal due to movement × susceptibility interaction, the six rigid-body-movement parameters, as determined from motion correction, were also included as covariates of no interest. Before fitting the model to the voxel time series, a high-pass filter with cut-off period at 128 sec was applied to the data. Finally, using a first-order autoregressive model, we removed serial correlations in our fMRI time series caused by aliased high-frequency physiological artifacts (e.g. cardiac-induced fluctuations). Then, model parameters were estimated by means of a Restricted Maximum-Likelihood (ReML) fit. Using the parameter estimates, linear contrast images were computed for the comparison vertical–horizontal wedges, the different ring eccentricities, the MT+ localizer, and the comparisons BFR–BR (i.e., [BFR1+BFR2]–[BR1+BR2]) as well as BFR–BF (i.e., [BFR1+BFR2]/2–BF).

*Individual retinotopic maps (Caret 5.51):* Individual retinotopic maps of visual areas V1 to V4 and area MT+ were obtained for each subject. To create the individual maps, gray and white matter were segmented in every subject. The segmented data were then inflated and flattened after removing topological errors [30]. The borders of the visual areas and the eccentricity intervals were defined for each subject, from the retinotopic mapping and the MT+ localizer. Thus, we defined ventral and dorsal areas V1, V2, V3, V3A, V4v, and area MT+. Regions corresponding to eccentricity intervals were defined within areas V1 to V4, where the line of maximum activity of a ring stimulus was taken as the border of an eccentricity interval. Region E1 represents the visual field within the inner ring, and regions E2 to E5 represent the fields between the five rings. Because of the logarithmic area change of retinotopic maps [20], [32] these regions of maximal activity are located slightly more laterally than the midline between the cortical projections of the inner and the outer stimulus radii. Their location was estimated by the equation 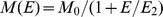 (with 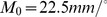 and 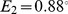) describing the dependency of the cortical magnification factor 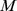 on eccentricity 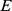 [20], [33], [34] with parameters obtained by us [35]. The eccentricity intervals were thereby estimated as E1: 0°–1.2°, E2: 1.2°–2.4°, E3: 2.4°–3.9°, E4: 3.9°–5.8°, E5: 5.8–8.5° (the stimulus for the paradigm on fusion and rivalry only extended to a maximum radius of 8.0°).

*Group statistics:* For group statistics, we extracted individual effect sizes of conditions within 41 visual areas (V1 to V4×dorsal/ventral×5 eccentricities + MT+) using the contrasts described above. These data were analyzed in two different ways: (1) a conventional analysis based on individual ROIs, (2) an alternative voxel-wise method using a functional maximum-probability map (MPM) of the visual cortex.

*(1) ROI based group analysis:* Using the transformation parameters from the cortical flattening process, voxels belonging to the certain visual areas were identified and combined into 41 ROIs. For each subject, first eigenvariates were extracted from these ROIs [36], [37], removing the variance of no interest (or *nuisance variance*).

*(2) Voxel-wise group analysis using a functional maximum-probability map of the visual cortex:* The probabilistic approach allows for a subsequent second-level analysis which goes beyond the standard method and can be expected to yield higher statistical power [38]–[40]. Here, we just sketch the method’s general outline; a detailed description is provided in Text S2.

First, a mean normalized structural image was used to create a group flat map, serving as a common reference system. Then, using individual contrast images for retinotopic mapping and the MT+ localizer, visual areas and eccentricity intervals were defined on the group flat map for each subject. Based on the ten resulting individual maps, maximum-probability ROIs were computed. Resulting ROIs comprised locations assigned to a certain visual area or eccentricity interval in the largest number of subjects and in at least half of the whole group.

For both approaches, three two-way analyses of variance (ANOVAs) were conducted in a ROI-based as well in a voxel-wise manner. Each of these ANOVAs contained the two-level factors CONDITION (comparisons BFR/BR, BFR/BF, and BR/BF respectively) and EYE (left/right). Random effects analyses were performed for effects as extracted from the individual ROIs as described in (1) and within a subset of voxels within the probabilistic retinotopic map of the whole group as described in (2). To identify these voxels, a voxel-wise 3×2 ANOVA for repeated measures with the factors CONDITION (BF/BR/BFR) and EYE (left/right) was performed in a first step. Voxels showing significant paradigm-associated BOLD effects were identified by F-testing the model at a liberal threshold of p<0.05. First eigenvariates of voxels within this subset of voxels were extracted from each probabilistic ROI for each condition. Then, *post-hoc* 2×2 ANOVAs for repeated measures were conducted for the eigenvariates of each ROI separately (i.e., 2×40 ANOVAs), again with the two-level factors CONDITION and EYE. F and *p* values were calculated for the two factors and their interaction. Results were regarded as significant if their *p*-value was p≤0.00125 (i.e. at the 5% level, Bonferroni-corrected for multiple testing with n = 40). To assess the direction of significant effects, *post-hoc* paired t-tests were computed. For these tests, the factor EYE was collapsed, i.e. the mean eigenvariates from BFR1 combined with BFR2 were tested against the values from BR1 combined with BR2 (or against condition BF); values from BR1 combined with BR2 were tested against the values from BF. A schematic depiction of the described data processing pipeline can be found in Figure S1.

## Results

### Brain responses on BF, BR and BFR

**Figure 2:**
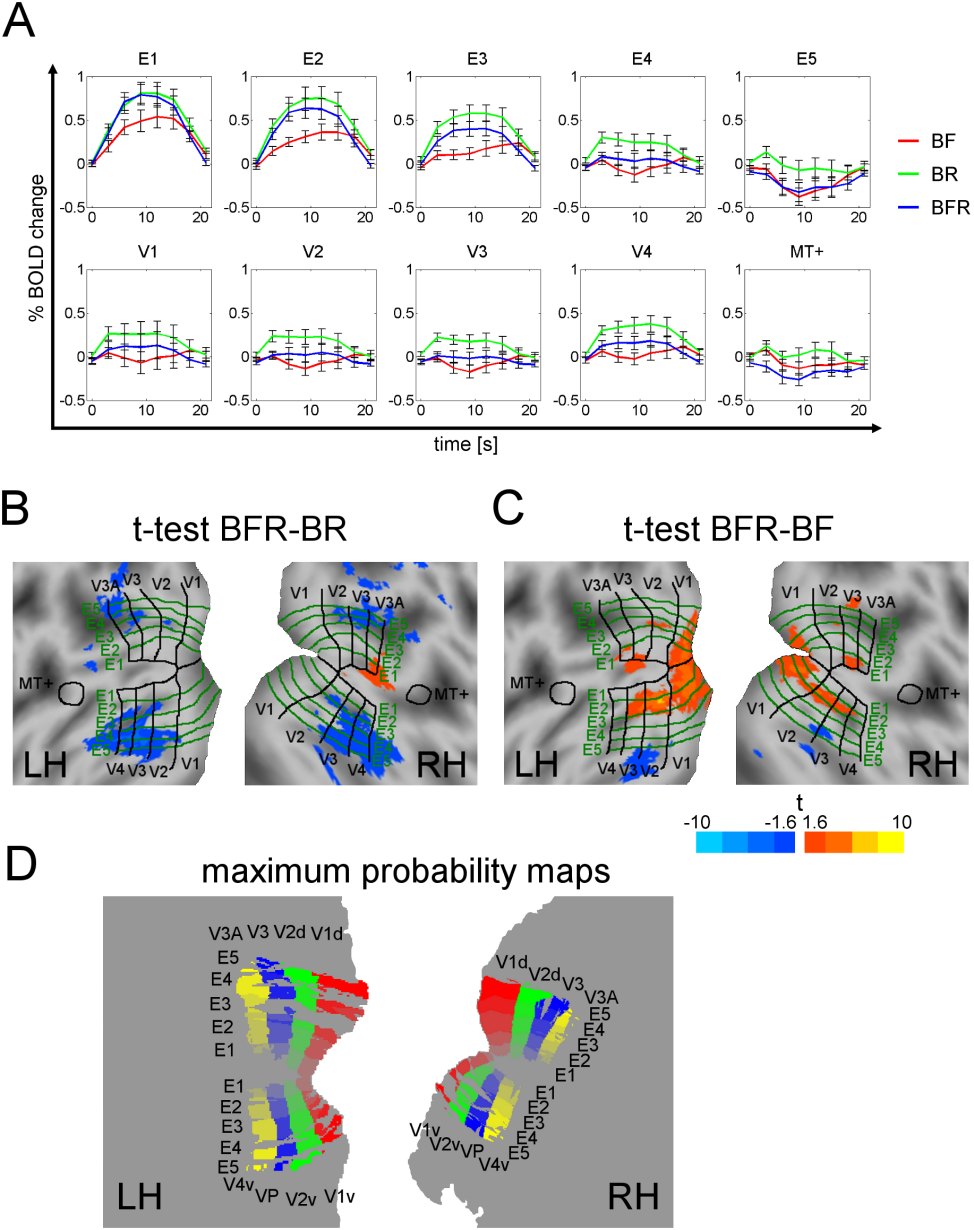
BOLD responses, individual flat map and maximum probability map for group analysis. (A) BOLD time series for the three conditions BF (red), BR (green) and BFR (blue), estimated for the individual visual areas (V1–V4 and MT+) and eccentricity intervals (E1–E5) within V1 to V4, averaged across all subjects (error bars: standard error of the mean). (B, C) Exemplary results on the individual flat map for a representative subject. The t-contrasts BFR/BR (B) and BFR/BF (C) are shown projected onto the flat maps of the subject’s left and right hemisphere (negative t-values: blue scale, positive t-values: red-to-yellow scale). Visual areas are separated by black lines and eccentricity intervals by green lines. (D) Maximum-probability flat maps (MPMs) of intersections of visual areas and eccentricity intervals for the subject group in normalized coordinates for the left and the right hemisphere. Areas are shown in different colors for better delineation (LH: left hemisphere, RH: right hemisphere).

Figure 2A shows average BOLD time series for the stimulus conditions BF, BR and BFR. The highest responses are observed for the rivalry condition BR, the lowest for the binocular-fusion condition BF. Within E1, the responses to BFR and BR are comparable. The BFR responses continuously decrease with increasing eccentricity, until reaching the same negative response level as in the fusion condition BF within E5. Furthermore, we found an overall decrease of signal amplitudes from E1 to E5 for all three conditions, which most likely arises from the stimulus-contrast decrease in the periphery [9], [41]. In the time series for the visual areas, the ratio between the values of BFR to BR and BF is similar within V1 to V4. While the overall signal changes are similar in V1 to V4, the lowest signal changes are found in area MT+. As BOLD responses in area MT+ were weak during all conditions, results for MT+ are not further considered.

### Evaluation on individual flat maps

To further resolve responses in subareas corresponding to different eccentricities, results of voxel-wise statistical tests were projected onto individual flat maps of the visual cortex. Figure 2B and 2C show color-coded t-values of the contrasts BFR–BR and BFR–BF, projected onto the flat maps of the left and right hemispheres of an exemplary subject. For a complete pattern, we provide the individual flat maps for all ten subjects in Figure S2. General tendencies can be observed in the two contrasts: in BFR–BR, the t-values tend to decrease from lower to higher eccentricities, and also from area V1 to V4. In contrast, for BFR–BF the effects rather increase for BFR compared to BF. Stronger effects are observed for lower eccentricities in earlier visual areas. Only in V3 and V4, the two conditions BF and BFR show similar response strengths.

### Group analysis of effects to visual fusion and binocular rivalry stimuli

Both the conventional method, based on individual ROIs, as well as the alternative voxel-wise method, yielded comparable results, with slightly stronger effects in the voxel-wise analysis. We thus restrict further reports on the results of the latter approach, providing the results of the ROI-based approach in the Supplement (Figure S3).

*Probabilistic retinotopic map of the group and MPM:* Figure 2D shows the MPM for intersections of visual areas (V1v, V1d, V2v, V2d, V3, VP, V3A, and V4v) with eccentricity intervals E1–E5, for the left and right hemisphere. Gaps in the outer eccentricity intervals E4 and E5 are caused by equal probabilities for neighboring regions and the resulting impossibility to assign to these certain areas.

### Main effect of the factor CONDITION

#### Comparison of BFR/BR

**Figure 3:**
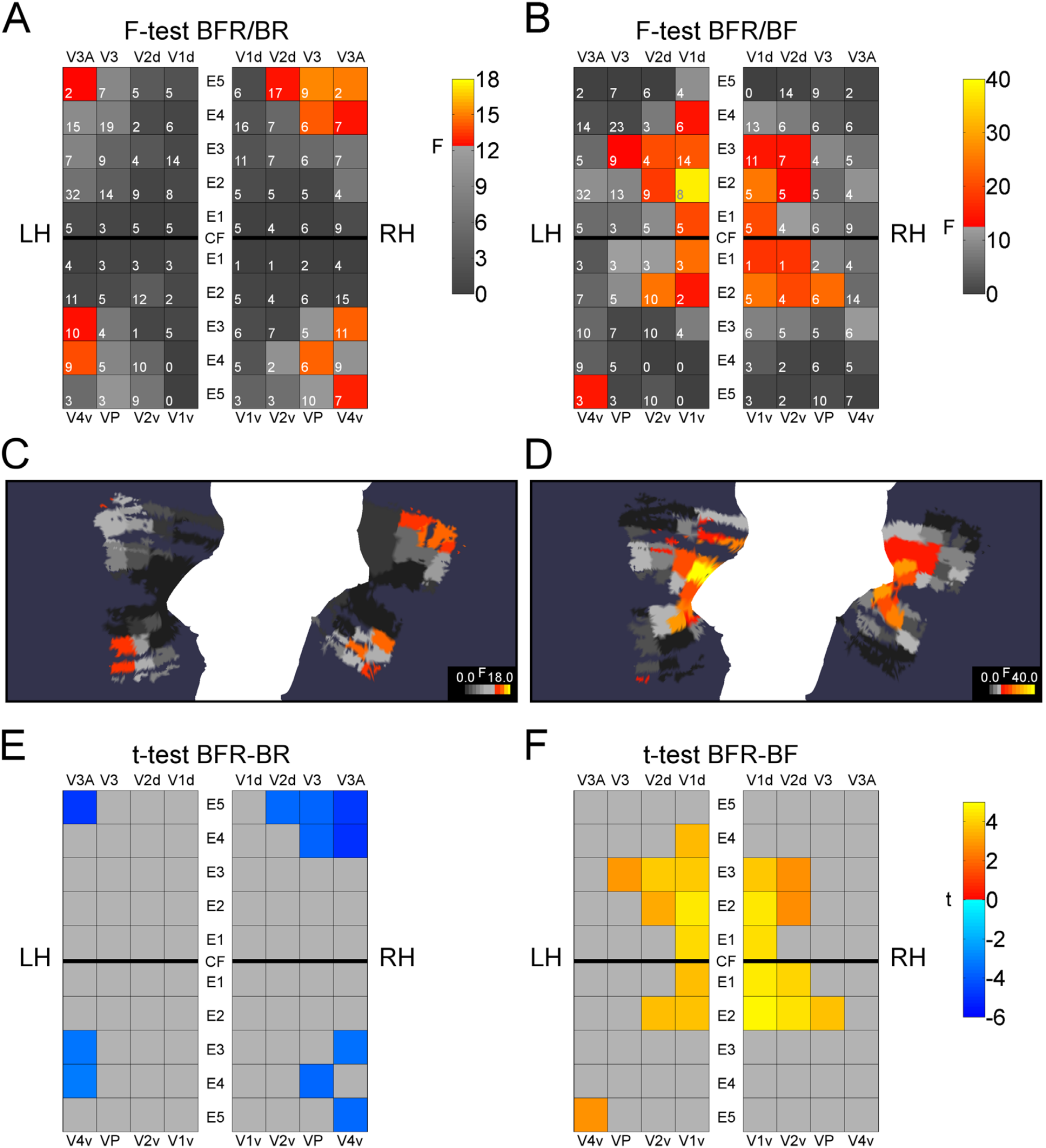
ROI-based evaluation results for conditions BFR and BR. F-test and t-test results are visualized for the 41 ROIs per hemisphere. Panels (A) and (C) show the results of the factor condition for the F-test for BFR/BR, and (B) and (D) show them for BFR/BF. ROIs for intersections of visual areas and eccentricity intervals (E1–E5) are arranged schematically in a pattern corresponding to flat maps of the visual cortex (LH: left hemisphere, RH: right hemisphere, CF: calcarine fissure) (A,B), or are shown as their masks projected on the flat map for the subject group (C, D). Significance level was set to 5%, Bonferroni corrected (p<0.05/40 = 0.00125). Significant ROIs are colored in a red-to-yellow scale, non-significant ROIs are shown with grey levels. The numbers within the ROIs in (A) and (C) denote the number of voxels that showed significant effects of stimulation (total volumes can be obtained by multiplying these with the voxel volume, V = 3×3×3 mm^3^). Note the different scale limits for F-values. Within intersection ROIs that showed significant effects of stimulation, a post-hoc paired t-test was performed. In panels (E) and (F), the t-values for BFR–BR and BFR–BF are visualized. The comparison BFR–BR (E) shows exclusively negative t-values for ROIs in areas V3/V4 at higher eccentricities. In BFR–BF (F) only positive t-values were found, mainly for ROIs in V1/V2 at lower eccentricities.

The effect sizes for the comparison BFR/BR increase from earlier to later visual areas, and from lower to higher eccentricities (Figures 3A and 3C). In the left hemisphere, three regions showed significant effects for the factor CONDITION, namely V4v/E4, V4v/E3, and V3A/E5 (see Table S1). In the right hemisphere, this was the case for eight regions, with the highest effects in V3/E5, V3A/E5 and VP/E4. No significant effects were found for eccentricities up to a radius of 2.4° (E1 and E2). The results of the *post-hoc* paired t-tests are shown in Figure 3E (and bottom part of Table S1). All *post-hoc* tests revealed higher responses in the condition BR. These finding was more pronounced for higher eccentricities, especially for areas V3, V3A, VP, and V4v.

#### Comparison of BFR/BF

Contrary to the results from the comparison of BFR/BR, regions with significant effects of BFR/BF were located mainly in areas V1 and V2 and at lower eccentricities (Figures 3B and 3D). This finding corresponds to the stimulus contrast decrease from the Gaussian envelope. In the left hemisphere, the largest effects were obtained in V1d/E2, V2v/E2 and V1v/E1, in the right hemisphere again in V1d/E2, V1v/E2, and also in VP/E2. For higher eccentricities, the only higher visual area with a significant effect was the left V4v/E5.

Results of the *post-hoc* paired t-test are shown in Figure 3F (and Table S1, middle column). *Post-hoc* tests revealed higher responses for the condition BFR. In the left hemisphere, the region with the largest response difference between BFR and BF were V1d/E2, V2d/E3 and V1d/E3; in the right hemisphere they were V1v/E2, V1d/E2, and V2v/E2. In summary, comparing the combined condition of fusible grid stimuli and an incompatible grid (BFR) to the condition of fusible grids (BF) resulted in effects mainly within intervals of lower eccentricities in areas V1 and V2, but not in V3 and V4.

#### Comparison of BR/BF

**Figure 4:**
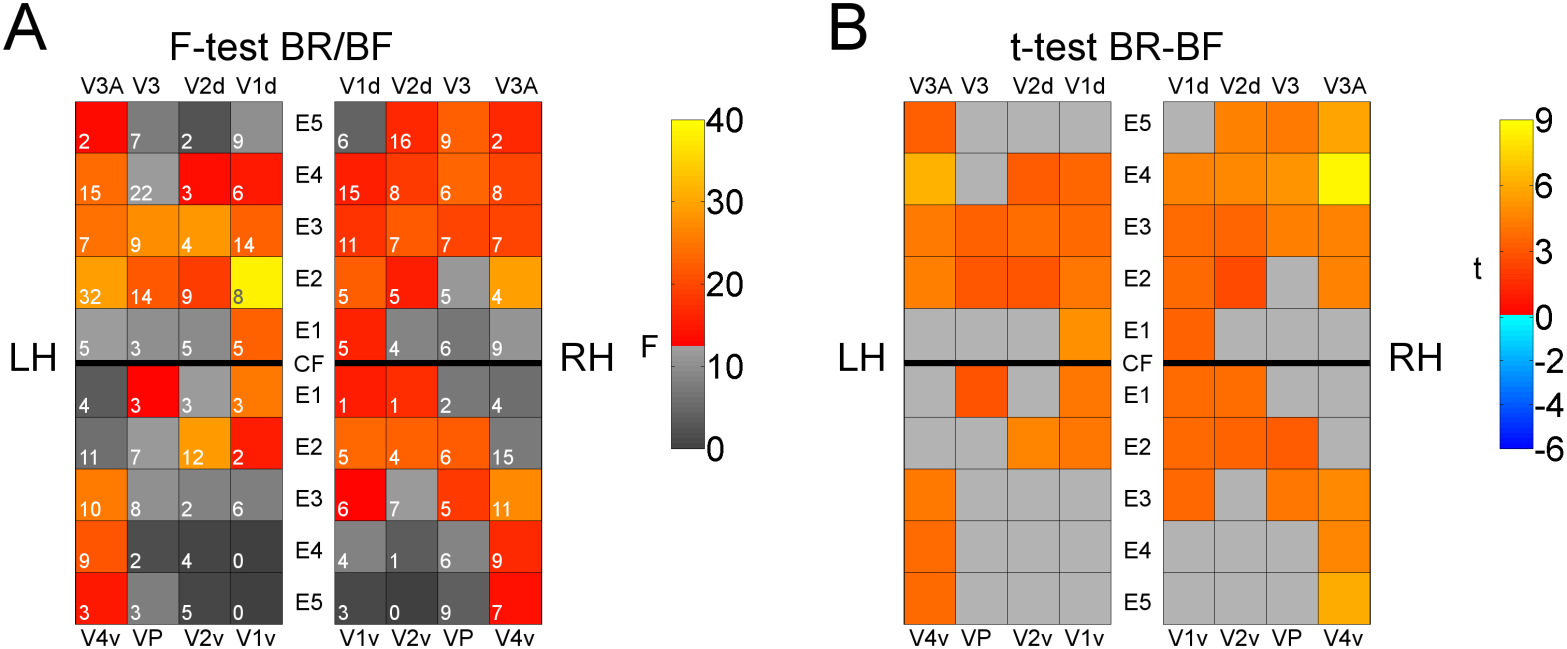
ROI-based evaluation results for the comparison of BR and BF. Visualizations of F-values (*A*) and t-values from the post-hoc paired t-test (*B*). As in the previous figure, ROIs for intersections of areas with eccentricity intervals are arranged in a pattern corresponding to flat maps of the visual cortex (LH: left hemisphere, RH: right hemisphere, CF: calcarine fissure). In panel (*A*) the numbers of voxels showing significant effects-of-interest that were included in evaluations are documented as white numbers.

In the left hemisphere, the largest differences between brain response to BR and BF were obtained in regions V1d/E2, V3A/E2, and V2v/E2. In the right hemisphere, regions V3A/E2, V4v/E3, and V1v/E2 showed the most pronounced differences (Figure 4A). In all regions, rivaling stimuli (BR) evoked stronger brain responses than fusible stimuli (BF).

### Main effect of EYE

The factor EYE did not show significant effects in any of the three comparisons.

### Interaction CONDITION × EYE

A single region showed an interaction between the factors CONDITION and EYE in the comparison BFR/BR (left-hemispheric V3/E5, F = 12.5, p = 0.0012). In BFR/BF and BR/BF, no significant interactions were found.

### Summary of Results

Our results can be summarized in three main findings: (1) Comparison of BR/BF shows stronger responses to the rivaling than to the fused grids in V1 to V4. (2) In BFR/BR, an additional fusible grid within rivaling ones is accompanied by a decrease of effects, especially in visual regions corresponding to higher eccentricities in V3/V4, and is absent in V1/V2. (3) In BFR/BF, an additional incompatible monocular stimulus within fusible grid stimuli leads to an increase of effects in V1/V2 but not in higher areas.

## Discussion

To investigate the differential neural processing of binocular information in the presence or absence of visual fusion, we designed stimulation conditions for binocular fusion (BF), binocular rivalry (BR), and an intermediate condition (BFR). The latter (BFR) includes the physical elements of BF and BR, but enables fusion as well as integration of incompatible monocular stimulus elements, without perceptual alternations.

In line with findings of Lee and Blake [10] and Moradi and Heeger [11], our study confirmed higher responses in V1/V2 and V3/V4 for rivaling compared to fused grids (BR–BF, Fig. 2A and 4B). Strikingly, our stimulus condition BFR had a differential effect on lower vs. higher areas: adding a monocular grid to fusible grids (when replacing BF by BFR) caused higher responses in the *earlier* areas V1/V2, predominantly at *lower* eccentricities. Removing the fusible grid from BFR (when replacing BFR by BR) caused an increase of responses in the *later* areas, V3/V4, at *higher* eccentricities (Figure S3). The higher responses for BFR compared to BF in areas V1 and V2 can be attributed to the additional monocular grid in the stimulus.

In the study by Moradi and Heeger [11], superimposing *fusible* gratings with further binocular stimulus content increased BOLD responses to a similarly extent in V1 to V4. In contrast, we found nearly unchanged responses in V3 and V4 when adding monocular stimulus content in the presence of fusion. We conclude that, during fusion, early areas V1 and V2 rather than higher areas V3 and V4 are involved in the processing of the monocular stimulus content.

### Possible origins of lower responses in BFR than BR

In common vision, monocular information is integrated into a fused percept. In our condition BFR, the remaining incompatible grid is also processed as compatible and therefore likely plays a different role than does the same grid in BR. The two conditions of fusion, BFR and BF evoke lower responses than the rivalry condition BR in areas V3 and V4, particularly at higher eccentricities, which may reflect a different aspect of visual fusion: Possible regulatory interactions between different types of binocular neurons depend on visual area and eccentricity. Single-cell experiments revealed classes of tuned-zero (T0) neurons that respond to zero disparity, and neurons tuned to larger disparities (near or far: TN,TF) [42], [43]. Tuned-zero neurons are thought to signal the horopter and maintain single vision by an inhibition of neurons tuned to different disparities. Neurons of the “near” or “far” system were shown to also respond to uncorrelated (as in rivalry) or monocular stimuli, although these responses were weaker than to stimuli of their favored disparity. Tuned-zero neurons are not active during rivalry and thus will not inhibit neurons tuned to larger disparities [43]. Along that line, in the case of rivalry, one can expect larger responses from classes of neurons that, in the absence of fusion, respond more broadly to monocular information. On that basis, one would expect higher neuronal responses to a rivalry (BR) than to a fused (BF, BFR) stimulus, since, in rivalry, inhibition from neurons signaling the horopter, is absent. The condition BFR, composed from fused upright grids and a monocular oblique grid, contains the same information of upright and oblique grids as BR, but enforces visual fusion. In that case, one would expect an inhibitory effect from neurons that signal the horopter. Therefore, unspecific responses to the monocular oblique grid in the rivalry condition BR should be reduced when adding fusible grids in BFR that constrain the responses to the oblique monocular grid. Taken together, the framework of specialized classes of binocular neurons provides a rationale for the observed higher BOLD responses to the rivalry stimulus BR than to the stimulus BFR (Figure 3E).

We further found a decrease in BOLD response in areas V3 and V4 at higher eccentricities, which leads to the question whether this pattern might correlate with distributions of those types of binocular neurons. It is known that, from V1 to V4, the fraction of TN and TF neurons increases, while the fraction of monocular neurons decreases. It is further known that the ratio between disparity-sensitive to disparity-insensitive cells (“flat cells”) strongly increases from V1 to V3/V3A (from a ratio of 1∶1 to 4∶1) [42]. Therefore, the fraction that would respond more broadly to a rivalry stimulus and is inhibited during fusion would increase from V1 to V4. Moreover, the known decrease of monocular neurons from V1 to higher areas would provide a rationale for the observed increase of responses in V1 and V2 for BFR compared to BF, as the stimulus BFR represents the stimulus BF with an additional monocular grid. Thus, attributing the observed pattern of BOLD response differences between BFR and BR to an active inhibitory mechanism in visual fusion would be in line with known properties and spatial distributions of different types of functional neuron populations, as proposed by Poggio et al. or Joshua and Bishop [42], [44].

Another framework for interpreting our findings could be the model recently proposed by Said and Heeger [45]. It draws upon the concept of redundancy reduction in stereo coding, which assumes that the signals of the two eyes are combined in such a way as to eliminate inter-ocular correlations of disparity information [46]. Said and Heeger’s model suggests that a class of neurons, which are active during rivalry, but inactive during fusion, is required to explain the efficient suppression during binocular rivalry [45]. This role is subserved by opponency neurons, originally proposed for stereo coding, which compute the difference in the signals between the two eyes. In the model, opponency neurons that are activated by the dominant stimulus inhibit monocular neurons that respond to the suppressed stimulus.

In our experiments, we instructed the subjects to fixate a cross in the center of the stimulus images, but did not track eye movements. This procedure is common in visual fMRI studies even though improper fixation represents a possible source of error. However, the meridian and eccentricity mapping in our subjects allowed a reliable definition of borders between visual areas and eccentricity intervals, suggesting that possible effects from unstable fixation are minor. In the worst case, deviations would have caused false negative results. In two recent fMRI studies, additional eye tracking experiments were performed outside the scanner to assess possible effects due to incorrect fixation. These experiments confirmed that the effects from eye-movement errors were negligible [47], [48].

### Cognitive Factors

Fusion and rivalry are conscious percepts, and in their formation, cognitive factors will play a role. Thus, correlates of the sensory processing in conditions of fusion or rivalry might be confounded with those from perceptual changes. We thus briefly consider the influence of attention and of conscious perception on our results.

Generally, attention has a modulatory effect on both neural activity and percepts. It is well known, that the reduction of selective, covert spatial attention reduces activity in the respective retinotopic areas [49]. Thus, diversion of spatial attention away from the stimulus influences the spatiotemporal dynamics of fMRI responses to a rivalry stimulus and modestly reduces their amplitude [20], [50]. A more special role of spatial attention for binocular rivalry was described by Zhang et al. [51]; by means of an EEG frequency-tagging method, they found that diverting attention away from rivaling images can stop rivalry and lead to neural representations of the dichoptic images being combined.

In our experiment, subject instructions to fixate the central cross were the same in all conditions. Thus, sustained attention [52] was likely centered at the fixation cross equally across conditions, and fMRI responses should not be affected by the locus of the sustained attentional spotlight. However, we cannot exclude that, from the rivalry percept in BR, transient attention might have been diverted away. This, in turn, could cause less stable rivalry in condition BR [15] and could have led to the observed effects in V3/V4. The rivalry condition might also have led to an overall increased level of attention, in turn leading to an overall increased level of activity. The question thus arises whether perhaps only because of attentional effects BR evoked higher responses than BFR. However, a comparison with the study by Moradi & Heeger [11] that used an attention diverting task shows that this is not likely. In their experiments, under diverted attention, rivaling gratings evoked higher responses than fused gratings in all observed visual areas with similar extent. In our study, BFR and BF evoked similar responses in V3/V4, and showed differences in V1/V2 only. These two fusible conditions should be similar with regard to attentional influences as they evoke a stable percept. Therefore, it is likely that even under diverted attention BFR shows lower responses than BR in areas V3 and V4, and that the observed effects of decreased responses in BFR relative to BR cannot be attributed to attentional effects.

## Conclusions

In summary, for distinguishing cortical mechanisms of visual fusion from those of rivalry, we introduced an intermediate stimulation condition that combines elements of fusion and of rivalry. Effects due to stimulus conditions for fusion and binocular rivalry were measured across eccentricities in areas V1 to V4. Compared to rivalry, additional fusible stimulus content led to lower responses in those subregions of areas V3 and V4 that correspond to higher eccentricities. Although the fusible content eliminated perceptual dichoptic suppression, cortical responses were decreased rather than increased in the peripheral regions of these later visual areas, suggesting that the circumstances of fusion caused this decrease. Our observations may be attributable to spatial distributions of different classes of neurons, causing inhibitory effects when changing from binocular rivalry to visual fusion. The earlier areas V1 and V2 thus show complementary behavior to V3 and V4, with respect to the roles of central and peripheral vision, in fusion and rivalry.

## Supporting Information

Figure S1 **Workflow of the main steps of data acquisition and evaluation.**
(PDF)

Figure S2 **Contrasts BFR–BR and BFR–BF on individual flat maps.** The results of the t-tests BFR–BR and BFR–BF were projected onto flat maps of each of the ten subjects.
(PDF)

Figure S3 **Evaluation based on individual retinotopic maps.** An evaluation of stimulus-evoked responses based on individual retinotopic maps shows similar results to that with maximum probability maps.
(PDF)

Table S1 **Results of F- and t-tests for comparisons between the conditions BFR, BR and BF.**
(DOCX)

Text S1 **Stimulus design – psychophysical considerations.**
(PDF)

Text S2 **Group analysis based on retinotopic maximum probability maps.**
(PDF)
