# Supplementary material for: Separating Fusion from Rivalry
Source: PLoS One. 2014 Jul 23;9(7):e103037. doi: 10.1371/journal.pone.0103037 (PMC4108392; doi:10.1371/journal.pone.0103037)
Supplement: Figure S1 — Workflow of the main steps of data acquisition and evaluation. (PDF) [file pone.0103037.s001.pdf]

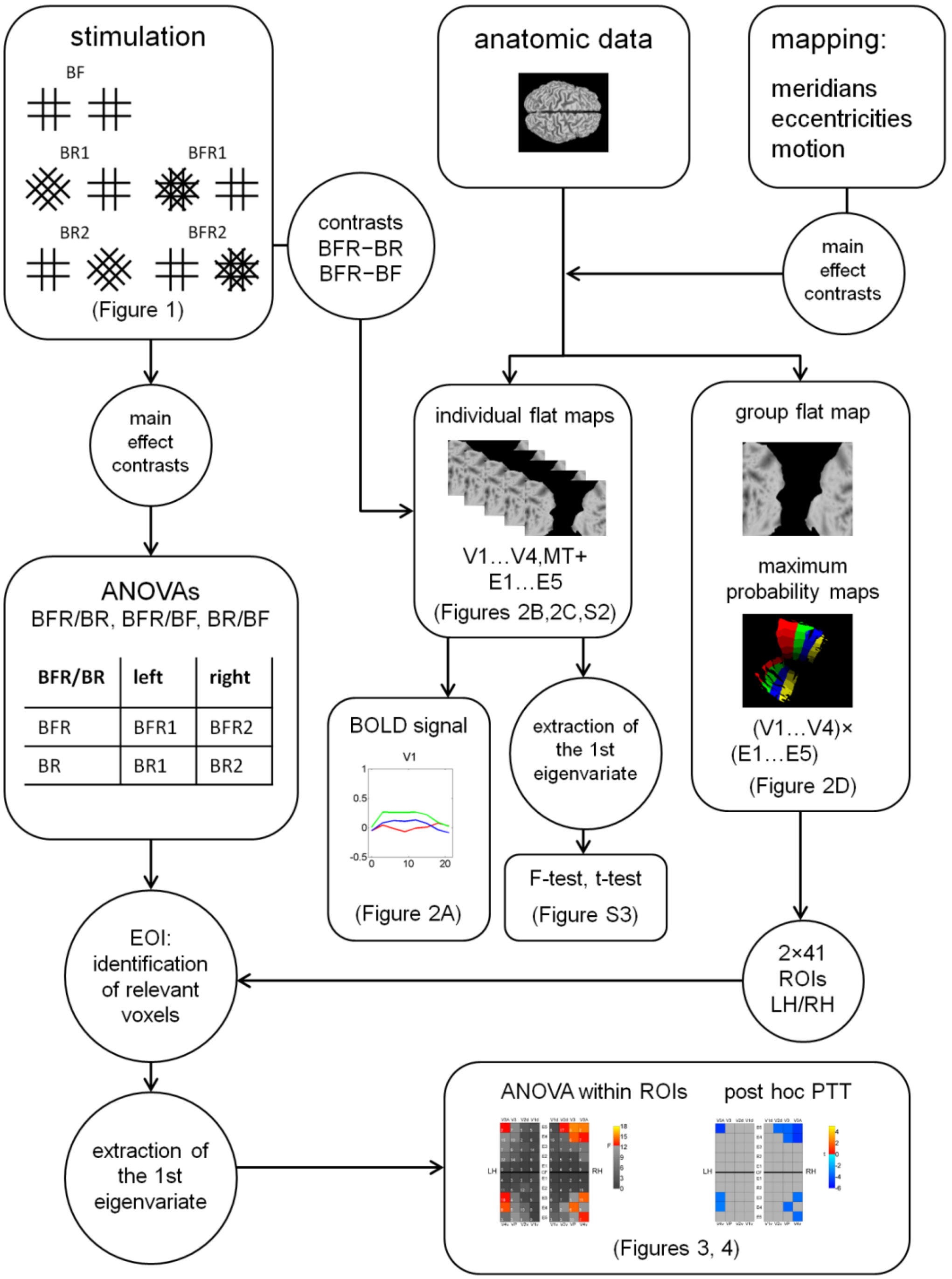

Figure S1. Workflow of the main steps of data acquisition and evaluation. References to the figures from the Results section are indicated in the illustrations of the corresponding workflow steps. Anatomical and functional data for mapping of visual areas and eccentricity intervals were used to define regions of interest (ROIs) on individual flat maps and on the group flat map. ROIs on the individual flat maps were used to extract BOLD-signal time series. To compare the effects of stimulation conditions on the individual flat maps, the contrasts were defined between BF (fused grid patterns), BR (rivaling grids) and BFR (fused grids with an added incompatible grid in either the left or the right eye). Individual linear-contrast images were calculated between one out of the five stimulation conditions and all others. Using these main effect contrasts, in a first ANOVA the levels of the two factors *condition* and *eye* were modeled in an "effects of interest" F-contrast (EOI) to identify relevant voxels that show effects to any of the stimuli. Inside each ROI, further evaluation was carried out in those voxels showing a significant EOI. The eigenvariate for significant voxels within a ROI was then extracted to calculate a post-hoc ANOVA. In ROIs showing significant effects, a post-hoc paired t-test (PTT) was calculated to evaluate the direction of effects.
