# Supplementary material for: Separating Fusion from Rivalry
Source: PLoS One. 2014 Jul 23;9(7):e103037. doi: 10.1371/journal.pone.0103037 (PMC4108392; doi:10.1371/journal.pone.0103037)
Supplement: Figure S2 — Contrasts BFR–BR and BFR–BF on individual flat maps. The results of the t-tests BFR–BR and BFR–BF were projected onto flat maps of each of the ten subjects. (PDF) [file pone.0103037.s002.pdf]

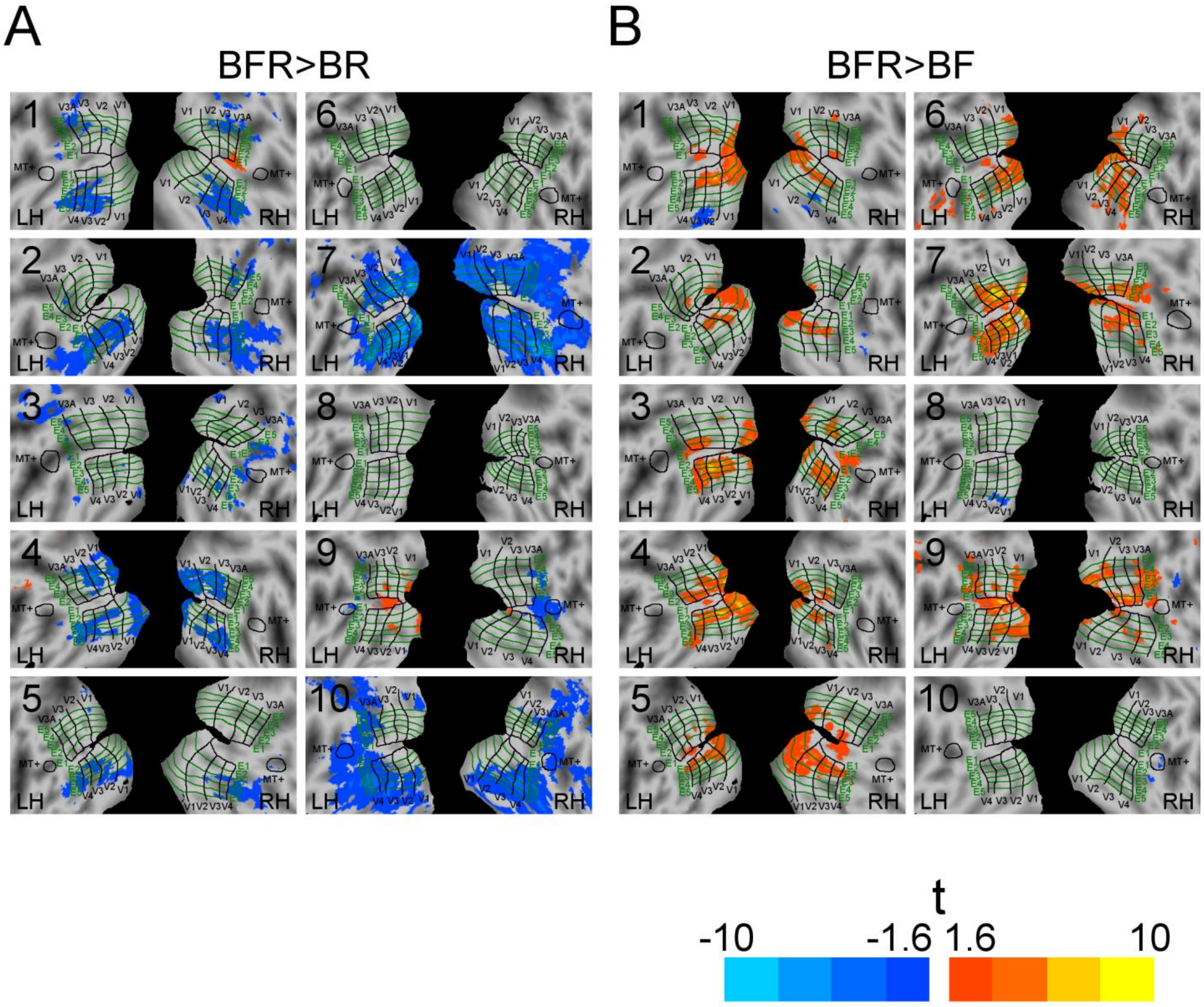

Figure S2. Contrasts BFR–BR and BFR–BF on individual flat maps. The results of the t-tests BFR–BR *(A)* and BFR–BF *(B)* were projected onto flat maps of each of the ten subjects (LH: left hemisphere; RH: right hemisphere). Significant t-values (p<0.05) are shown color-coded in blue (negative values) and yellow/red (positive values). Visual areas V1 to V4 and MT+ (black lines), as well as eccentricity intervals E1 to E5 (green lines), were drawn according to the results of the retinotopic mapping. The individual flat maps were created from non-normalized anatomical data. The effect strengths and localizations were variable across the ten subjects. Nevertheless, general tendencies can be observed. In BFR–BR *(A),* t-values are mainly negative, tend to decrease from lower to higher eccentricities, and to decrease with ascending area from V1 to V4. In contrast, BFR–BF *(B)* shows positive t-values, with larger values at lower eccentricities within V1 and V2.
