# Supplementary material for: Separating Fusion from Rivalry
Source: PLoS One. 2014 Jul 23;9(7):e103037. doi: 10.1371/journal.pone.0103037 (PMC4108392; doi:10.1371/journal.pone.0103037)
Supplement: Figure S3 — Evaluation based on individual retinotopic maps. An evaluation of stimulus-evoked responses based on individual retinotopic maps shows similar results to that with maximum probability maps. (PDF) [file pone.0103037.s003.pdf]

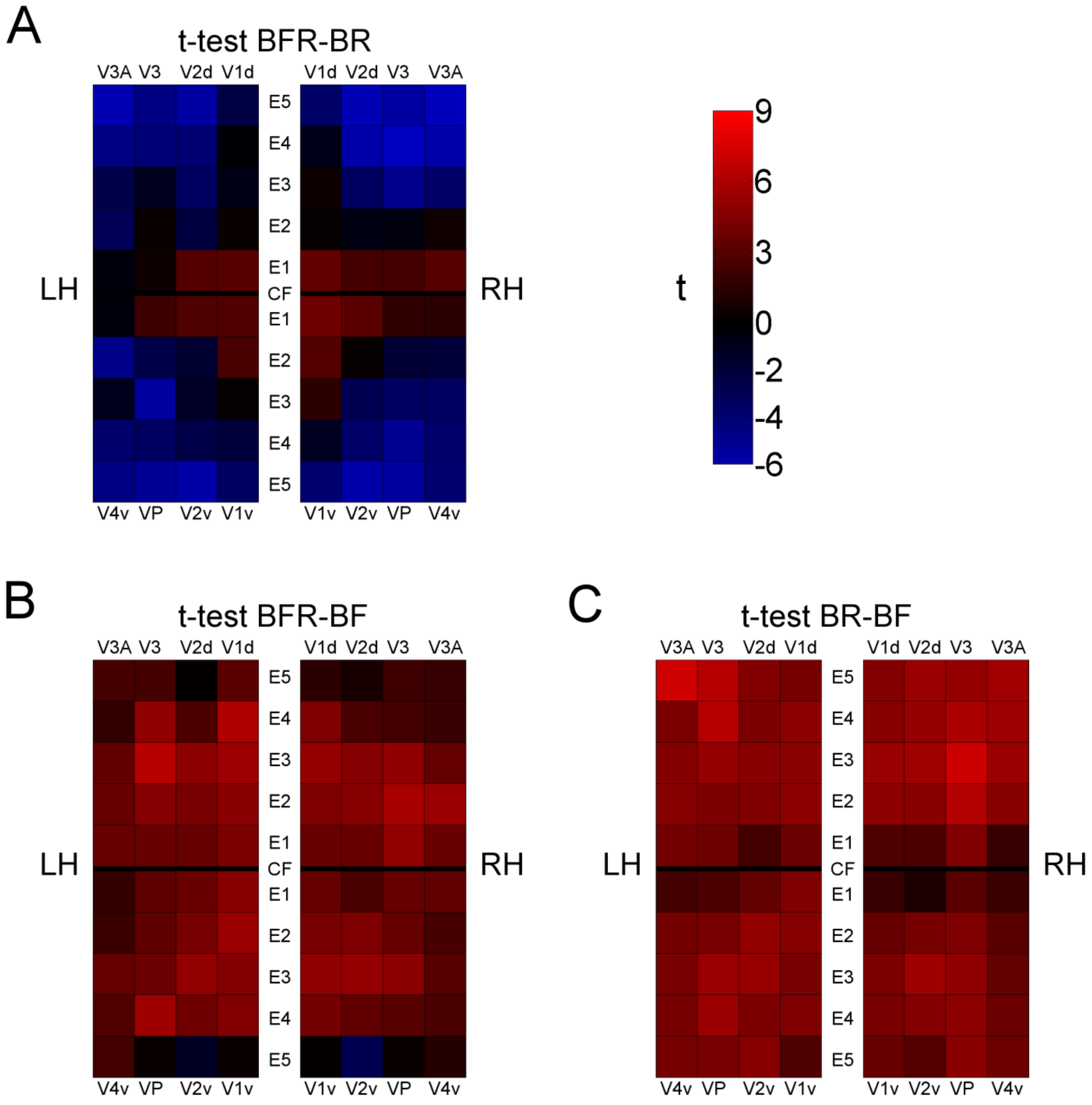

Figure S3. Evaluation based on individual retinotopic maps. An evaluation of stimulus-evoked responses based on individual retinotopic maps shows similar results to that with maximum probability maps. *(A-C)* Color coded t-values for comparisons between stimulus conditions are shown for eccentricity regions in areas V1 to V4, similar to that in Figures 3 and 4 (LH: left hemisphere, RH: right hemisphere, dorsal areas: V1d–V3A, ventral areas: V1v–V4v, eccentricity intervals: E1–E5, CF: calcarine fissure). For each subject, areas and eccentricity intervals were defined on flat maps created from individual anatomical data. Within each ROI of intersections between areas and eccentricity intervals, first eigenvariates were extracted for the stimulus conditions BF, BR1, BR2, BFR1 and BFR2, respectively. Panel *(A)* shows t-test results for the test BFR–BR (BFR1+BFR2 against BR1+BR2). BFR induced weaker responses than BR, to an increasing extent from V1 to V4 as well as with increasing eccentricity, similar to the corresponding evaluation in probabilistic areas (see Figure 3). At lower eccentricities, preferentially in areas V1 and V2, BFR induced stronger responses than BR, probably due to the increase in monocular stimulus content in BFR (rivaling grids plus monocular upright grid) compared to BR (rivaling grids). Panel *(B)* shows t-test results for the test BFR–BF ([BFR1+BFR2]/2 against BF). BFR induced stronger responses than BF, to a decreasing extent from lower to higher eccentricities and from V1 to V4, similar to responses in Figure 3. Panel *(C)* shows t-test results for BR–BF ([BR1+BR2]/2 against BF). BR induced stronger responses than BF in areas V1 to V4 in lower and higher eccentricity regions.
