# Supplementary material for: Separating Fusion from Rivalry
Source: PLoS One. 2014 Jul 23;9(7):e103037. doi: 10.1371/journal.pone.0103037 (PMC4108392; doi:10.1371/journal.pone.0103037)
Supplement: Table S1 — Results of F- and t-tests for comparisons between the conditions BFR, BR and BF. (DOCX) [file pone.0103037.s004.docx]

Table S1. Results of F- and t-tests for comparisons between the conditions BFR, BR and BF. Results of statistical tests in the five most significant intersection ROIs of visual areas and eccentricity intervals are given (in BFR/BR only three ROIs were significant); the two panels show F-tests for the factor *condition* (top), and post-hoc paired t-tests (bottom) (LH: left hemisphere, RH: right hemisphere). While the comparison BFR–BR (t-test) shows significant decreases of responses for ROIs in V3/V4 at higher eccentricities, BFR–BF shows significant increases mainly in V1/V2 at lower eccentricities. The comparison between BR and BF (on the right) includes both tendencies from BFR/BR and BFR/BF.

| **BFR/BR (ANOVA)** | | | **BFR/BF (ANOVA)** | | | **BR/BF (ANOVA)** | | |
| --- | --- | --- | --- | --- | --- | --- | --- | --- |
| ***LH*** | | | ***LH*** | | | ***LH*** | | |
| ***ROI*** | ***F*** | ***p*** | ***ROI*** | ***F*** | ***p*** | ***ROI*** | ***F*** | ***p*** |
| V4v/E4 | 14.0 | 0.0006 | V1d/E2 | 38.3 | 3.9·10^-7^ | V1d/E2 | 38.6 | 3.6·10^-7^ |
| V4v/E3 | 12.6 | 0.0011 | V2v/E2 | 24.8 | 1.6·10^-5^ | V3A/E2 | 29.4 | 4.1·10^-6^ |
| V3A/E5 | 12.4 | 0.0012 | V1v/E1 | 24.2 | 1.9·10^-5^ | V2v/E2 | 29.0 | 4.6·10^-6^ |
|  |  |  | V1d/E3 | 21.5 | 4.5·10^-5^ | V2d/E3 | 28.7 | 4.9·10^-6^ |
|  |  |  | V2d/E3 | 20.6 | 6.1·10^-5^ | V3/E3 | 27.6 | 6.8·10^-6^ |
| ***RH*** |  |  | ***RH*** |  |  | ***RH*** |  |  |
| ***ROI*** | ***F*** | ***p*** | ***ROI*** | ***F*** | ***p*** | ***ROI*** | ***F*** | ***p*** |
| V3/E5 | 15.3 | 0.0004 | V1d/E2 | 25.3 | 1.4·10^-5^ | V3A/E2 | 29.5 | 4.0·10^-6^ |
| V3A/E5 | 15.1 | 0.0004 | V1v/E2 | 24.7 | 1.6·10^-5^ | V4v/E3 | 27.2 | 7.7·10^-6^ |
| VP/E4 | 14.5 | 0.0005 | VP/E2 | 23.8 | 2.2·10^-5^ | V1v/E2 | 23.6 | 2.3·10^-5^ |
| V4v/E3 | 14.4 | 0.0005 | V1d/E1 | 20.3 | 6.7·10^-5^ | V2v/E2 | 22.7 | 3.0·10^-5^ |
| V3/E4 | 14.3 | 0.0006 | V2v/E2 | 20.0 | 7.5·10^-5^ | V3/E4 | 23.3 | 2.6·10^-5^ |

| **BFR**–**BR (post hoc PTT)** | | | **BFR**–**BF (post hoc PTT)** | | | **BR**–**BF (post hoc PTT)** | | |
| --- | --- | --- | --- | --- | --- | --- | --- | --- |
| ***LH*** |  |  | ***LH*** |  |  | ***LH*** |  |  |
| ***ROI*** | ***t*** | ***p*** | ***ROI*** | ***t*** | ***p*** | ***ROI*** | ***t*** | ***p*** |
| V3A/E5 | -4.72 | 0.0011 | V1d/E2 | 4.57 | 0.0014 | V2v/E2 | 4.69 | 0.0011 |
| V4v/E3 | -3.25 | 0.0100 | V2d/E3 | 3.97 | 0.0032 | V3A/E2 | 4.52 | 0.0014 |
| V4v/E4 | -3.14 | 0.0125 | V1d/E3 | 3.96 | 0.0033 | V1d/E2 | 4.16 | 0.0025 |
|  |  |  | V1v/E1 | 3.73 | 0.0047 | V2d/E3 | 3.85 | 0.0039 |
|  |  |  | V2v/E2 | 3.73 | 0.0047 | V3/E3 | 3.43 | 0.0075 |
| ***RH*** |  |  | ***RH*** |  |  | ***RH*** |  |  |
| ***ROI*** | ***t*** | ***p*** | ***ROI*** | ***t*** | ***p*** | ***ROI*** | ***t*** | ***p*** |
| V3A/E5 | -4.71 | 0.0011 | V1v/E2 | 4.83 | 0.0009 | V3/E4 | 5.17 | 0.0006 |
| V3/E4 | -3.70 | 0.0049 | V1d/E2 | 4.55 | 0.0014 | V4v/E3 | 4.83 | 0.0009 |
| V3/E5 | -3.64 | 0.0054 | V2v/E2 | 4.45 | 0.0016 | V3A/E2 | 4.57 | 0.0013 |
| VP/E4 | -3.61 | 0.0057 | V1d/E1 | 4.37 | 0.0018 | V1v/E2 | 3.72 | 0.0048 |
| V4v/E3 | -3.36 | 0.0084 | VP/E2 | 3.79 | 0.0043 | V2v/E2 | 3.50 | 0.0068 |
