# Supplementary material for: Separating Fusion from Rivalry
Source: PLoS One. 2014 Jul 23;9(7):e103037. doi: 10.1371/journal.pone.0103037 (PMC4108392; doi:10.1371/journal.pone.0103037)
Supplement: Text S1 — Stimulus design – psychophysical considerations. (PDF) [file pone.0103037.s005.pdf]

Supporting Information Text S1
Stimulus design – psychophysical considerations

After switching off rivalry, the state of fusion does not occur instantaneously [1–3]. We took particular care to allow fast switching: In Liu et al.'s study, lag time until the onset of rivalry was increased to several seconds at low contrasts and higher fundamental spatial frequencies. It further depended on waveform, where waveforms with more high-frequency content needed higher contrast to quickly elicit rivalry. However, at *low* (fundamental) spatial frequency (below 2 cpd), lag time was low (as desired), even at low contrasts (below 10%; Fig. 5 in Liu et al. [2]). The study further showed that with missing fundamental – which is close to our situation with thin lines – and with low fundamental spatial frequency as in our case, much more contrast is needed than with full waves to elicit rivalry at 2 sec duration. Around 15% peak-to-peak contrast is needed (Fig. 6 in Liu et al. [2]). In our stimuli, we have a fundamental spatial frequency between 1.25 and 1.8 c/deg and a (peak-to-peak) contrast of the lines of 33% in the center, decreasing below 10% at around 5.8° eccentricity, which is for the most part higher than required. Thus, very short lags would be expected for rivalry setting in. This prediction was confirmed in our pilot experiments in which button presses and verbal reports indicated that the lag time of rivalry onset was reliably small, i.e. less than a second. FMRI block time, in comparison, is 16 sec. Therefore, our condition BR can validly serve as a rivalry condition.

References

1. Julesz B, Tyler CW (1976) Neurontropy, an entropy-like measure of neural correlation, in binocular fusion and rivalry. Biol Cybern 23: 25–32.

2. Liu L, Tyler CW, Schor CM (1992) Failure of rivalry at low contrast: evidence of a suprathreshold binocular summation process. Vision Res 32: 1471–1479.

3. Tyler CW, Julesz B (1976) The neural transfer characteristic (neurontropy) for binocular stochastic stimulation. Biol Cybern 23: 33–37.
