# Supplementary material for: Separating Fusion from Rivalry
Source: PLoS One. 2014 Jul 23;9(7):e103037. doi: 10.1371/journal.pone.0103037 (PMC4108392; doi:10.1371/journal.pone.0103037)
Supplement: Text S2 — Group analysis based on retinotopic maximum probability maps. (PDF) [file pone.0103037.s006.pdf]

Supporting Information Text S2
Group analysis based on retinotopic maximum probability maps

To evaluate our paradigm on visual fusion and binocular rivalry, we conducted group analyses following two different approaches. On the one hand, a conventional analysis based on individual retinotopic maps was performed. On the other hand, to improve filtering for relevant effects and obtain higher statistical power, we designed an alternative approach based on probabilistic ROIs for the subject group with a subsequent second-level analysis.

For this purpose, a group flat map was created from the normalized (MNI space) anatomical data to obtain a common reference system for the individual maps. Gray and white matter were first segmented in all subjects. Voxels that belonged to gray matter in at least half of the subjects were assigned to the group gray matter using MRIcron (Center for Advanced Brain Imaging, Atlanta GA, USA), resulting in a median anatomic gray-matter volume. These data were then inflated and flattened after correcting topological errors. On these anatomical maps, we created functional, retinotopic maps for each subject in the normalized coordinates of the group gray-matter flat map. In these maps, visual areas and eccentricity intervals were defined for every subject from the meridian mapping, respectively.

To create a representative functional map of the visual cortex for the whole group, the retinotopic maps from each of the ten subjects were superimposed onto the group gray-matter map. To obtain the probability of the surface element at a given position belonging to a certain area, area probabilities $p_a \in \{0;0.1;0.2\ldots1\}$ for every location were computed. These probabilities correspond to the number of subjects that had area a assigned to that location. These analyses (and all further described below) used custom scripts in MatLab (The Mathworks, Natick, MA, USA) written by the authors. A location was classified as belonging to a specific area of the MPM if the probability was highest for that area and also exceeded a threshold of $p_a = 0.5$. Eight cortical visual areas, located ventrally or dorsally from the calcarine fissure, were thus defined; these are V1v, V1d, V2v, V2d, V3, VP, V3A, and V4v. In the very same way, cortical regions corresponding to eccentricity intervals E1–E5 were defined. Now an MPM for eccentricities was created from the superimposed individual eccentricity maps by relating locations to eccentricity intervals of highest probability, again with $p_a \geq 0.5$ additionally required. The MPMs for visual areas and eccentricity intervals were finally superimposed to arrive at maps showing the intersections of the two boundary sets. This approach permits the use of standard statistical approaches as implemented in SPM5 (Wellcome Department of Imaging Neurosciences, London) – specifically the Gaussian random-field-based alpha-error adjustment and, additionally, the use of probabilistic retinotopic information.

Finally, to extract condition-related BOLD time series and to assess region specific activity, the retinotopic MPMs were converted to 8×5=40 regions of interest (ROIs). Surface element positions assigned to a certain visual area were back-transformed into individual voxel space. Thus, voxel masks were created for 40 ROIs in each hemisphere.
